# The effects of technology use on children's physical activity: a cross-sectional study in the Eastern province of Saudi Arabia

**DOI:** 10.25122/jml-2022-0148

**Published:** 2022-10

**Authors:** Abdullah Almaqhawi, Mohammed Albarqi

**Affiliations:** 1 Department of Family Medicine and Community, College of Medicine, King Faisal University, Al Ahsa, Saudi Arabia

**Keywords:** physical activity, technology use, screen time, children, youth, survey

## Abstract

Recently, a considerable amount of literature has been concerned with the impact of screen time on physical activity. Furthermore, recent evidence reveals that children under 14 spend an average of 23 hours each week looking at screens, including watching TV and DVDs, playing video games, or using a computer or mobile device. This study aimed to determine the relationship between technology use and physical activity. 277 parents completed an online questionnaire in this cross-sectional investigation. The questionnaire comprised 44 closed-ended questions divided into three sections: demographics, the impact of technology on children, and the Children's Physical Activity Questionnaire (CPAQ). 88 (31.8%) of children reported up to 5 hours of screen time per day, while 189 (68.2%) reported 6 hours or more. According to the CPAQ, 131 (47.3%) children had a low level of physical activity, 96 (34.7%) had a moderate level, and 50 (18.1%) had a high level. There was a strong relationship between parental age and child screen time, with 24.9% of children with screen time greater than 6 hours having parents aged 35–40 years, compared to 28.4% of children with screen time less than 5 hours having parents aged 25–30 years. Inadequate physical activity in children was linked to the number of siblings, ownership of electronic devices, and screen time. Physical activity should be increased through lifestyle changes that the entire family can implement.

## INTRODUCTION

Recently, there has been an increased interest in using various forms of technology. The use of cell phones and computers has expanded over the previous two decades, increasing reliance on these technologies for day-to-day work [1]. Children are among the most enthusiastic consumers of technology [2]. Data from Saudi Arabia's General Authority of Statistics (GAS) report that 98.44% of houses or families have televisions, 61.08% have computers, 83.87% have internet access, and 47.21% have smart devices [3]. It is difficult to predict if this shift in technology use will have a beneficial or harmful impact on human health at this time, especially with the current Covid-19 pandemic and the challenges associated with safe distance and remote learning. According to recent studies, the average Saudi toddler's screen use is approximately 3 hours per day (television for about 2 hours a day and mobile devices for about 1 hour a day) [4].

Furthermore, it was previously observed that electronic devices improve children's learning abilities, communication skills, language ability, analytical capacity, creative thinking, and extraction of both academic and non-academic activities [5]. However, several studies suggest that children who use gadgets regularly disregard their surroundings and remain isolated from nature and their environment [6–8]. Additionally, research shows that children who use devices for more than 4 hours each day become addicted to technology and exhibit negative behaviour in daily life [9].

The use of technology can contribute to poor health by increasing disease and reducing physical activity, particularly among children. Children who use technology are more likely to develop obesity, type 2 diabetes mellitus, all-cause mortality, metabolic syndrome, and various medical and psychological disorders [10–13]. Correspondingly, numerous studies have found that children who use technology have an increased risk of sleep anxiety, night waking, and complete sleep disruption [14, 15], in addition to increasing physiological, emotional, or mental arousal [16]. Increased reliance on technology has been linked to increased anxiety symptoms, sadness, irritability, and attention and behavioural issues [17].

The use of technology in children may directly or indirectly affect the level of physical activity. Cardiorespiratory fitness, obesity, the quality of physical education programs, TV consumption, and parental influence appear to be the major determinants of physical activity in Saudi children and adolescents [18]. Physical inactivity and sedentary behaviour are substantial risk factors for various noncommunicable diseases, including diabetes, obesity, cardiovascular disorders, and mental impairments [19]. According to Al-brief Hazzaa's review, the prevalence of physical inactivity among Saudi children and adults ranged from 43.3 to 99.5% [20]. Similarly, data from several studies suggest that Saudi Arabian people are not active enough to get the health benefits of physical activity [21, 22]. As a result, this study aimed to determine the association between technology use and physical activity and examine the relationship between sociodemographic factors in Saudi Arabian children aged 6–12.

## Material and Methods

This cross-sectional study used an online questionnaire to collect data on technology use and physical activity among children between 6 to 12 years living in the eastern region of Saudi Arabia. The questionnaire was developed based on modified questionnaires from a prior study [22]. A link was distributed using social media platforms (Twitter, Facebook, and WhatsApp) to gather the maximum number of participants from 12/04/2021 to 15/06/2021. Saudi Arabian parents were proxies responding on behalf of their children to fill out an anonymous survey. According to the computer software (EPI INFO) sample size calculator, the minimal sample size was 270 with a confidence level of 95% (sample size=Z2*(p)*(1-p)/c2). Based on the estimate of receiving 50% of the responses, the survey was distributed to over 550 parents. After applying exclusion criteria, 277 replies were obtained, representing a response rate of 50.4%. Parents answered a survey on demographics, technology use, and physical exercise in Arabic. The questionnaire has a section for giving informed consent and protecting the participants' privacy. The questionnaire reliability was verified using consultants' reviews and a pilot study of the first ten responses using Cronbach's alpha test. The questionnaire consisted of 44 close-ended questions divided into three sections [23, 24].

The first section was designed to collect demographic and education data for the parents, the child, and information regarding the family. The second section represented the Children's Physical Activity Questionnaire (CPAQ), where parents were asked to rate the type, frequency, and length of physical and sedentary activities, including school and leisure time, over the previous seven days. The first component of the questionnaire analysed seven-day activities outside school time, such as soccer, volleyball, tennis, swimming, basketball, and running. The following subsection assessed leisure activities such as bike riding, home chores, pet play, trampoline, scooter, playground equipment play, walking, or skipping rope. The response options were "yes" or "no", and the total amount of time spent on the activity during the week was stated in hours per week. The last section was the Impact of Technology on Children questionnaire designed for children aged 4 to 12 and included questions about the child's technology use, after-school activities, sleep patterns, behaviour, and emotions. Only questions about the child's technology use were used for this study. This section of the survey contained questions concerning the child's electronic device ownership, including the number and type/s of device/s possessed by the child, the age at which the child first acquired a device, the number of hours per day spent viewing television or DVDs and playing video games such as PS3^®^ (Sony Corporation, Tokyo, Japan), Xbox^®^ (Microsoft, Redmond, WA, USA), or Wii^®^ (Foxconn, New Taipei City, Taiwan) or playing internet games, playing on a portable games console and use of Facebook or other social networking sites, *e.g*., Twitter or Instagram.

Following data extraction, data were reviewed, coded, and entered into the statistical software program IBM SPSS version 22 (SPSS, Inc. Chicago, IL). Two-tailed tests were used for all statistical analyses. A p-value of less than 0.05 was considered statistically significant. For statistical purposes, technology consumption time was dichotomized using the median as a cut-off point: total hours spent on the internet, social media, video games, and portable devices were computed, and individuals were separated into two groups. Children who spent five hours or less on screens (screen-time ≤5 h) were classified as low screen-time users, while those who spent six hours or more on screens (screen-time ≥6 h) were classified as high screen-time users. Physical activities were divided into three groups for the CPAQ based on the metabolic equivalent of task (MET): light-intensity (MET 3), moderate-intensity (MET 3 to 6), and vigorous-intensity (MET>6). We performed descriptive statistics (frequency and percent distribution) for all variables, including parents' and children's demographic data, screen time use for different devices, physical activity using CPAQ, and children's electronic device ownership. The Pearson chi-square test was used to examine the distribution of students' screen time consumption, demographic data, and physical activity. A logistic regression model was utilised to determine the most significant determinants of inadequate physical activity among children.

## Results

**Figure 1:**
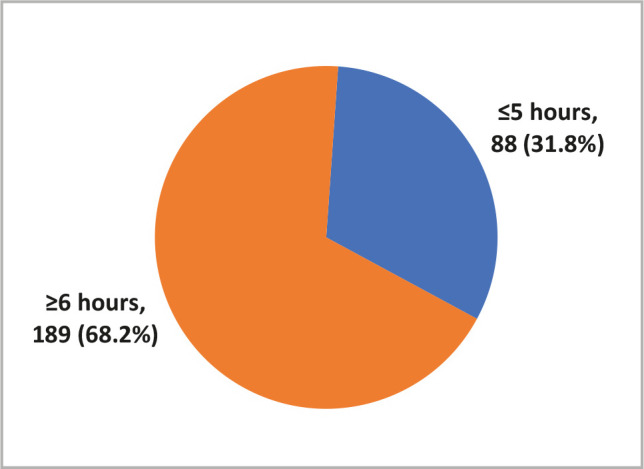
Screen time among Saudi children aged 6–12 years.

**Figure 2:**
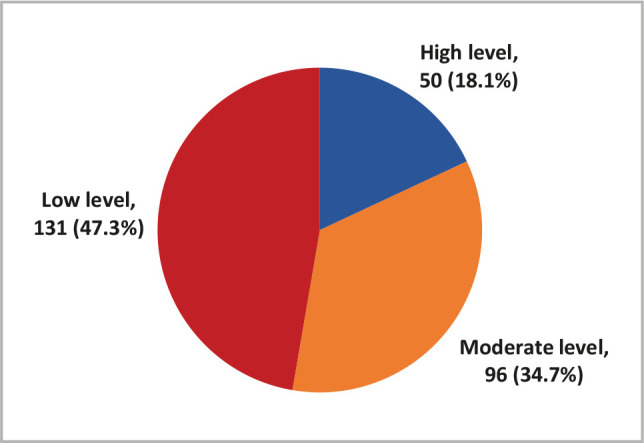
Physical activity based on CPAQ among Saudi children aged 6–12 years.

Data from 277 children were collected through their parents or caregivers. 88 (31.8%) children reported up to 5 hours of screen time daily, while 189 (68.2%) reported 6 hours or more (Figure 1). Based on the CPAQ, 131 (47.3%) children recorded a low level of physical activity, 96 (34.7%) recorded moderate level physical activity, and 50 (18.1%) recorded high-level physical activity (Figure 2).

There was a significant association between parents' age and children's screen time, with 24.9% of children with >6 hours of daily screen time having parents aged 35–40 years, and 28.4% of children with daily screen time <5 hours having parents aged 25–30 years (P=.021, Table 1). Also, 49.2% of children with screen time >6 hours had 2–3 siblings compared to 59.1% of those with screen time <5 hours (P=.001).

**Table 1:** Association between technology use and parents' demographic data.

Parent data	Total		Screen time				P-value
			≤5 hours		≥6 hours		
	No.	%	No.	%	No.	%	
**Age in years**							
25–30	49	17.7%	25	28.4%	24	12.7%	.021*
31–35	60	21.7%	19	21.6%	41	21.7%	
35–40	66	23.8%	19	21.6%	47	24.9%	
40–45	47	17.0%	10	11.4%	37	19.6%	
>45	55	19.9%	15	17.0%	40	21.2%	
**Marital status**							
Married	256	92.4%	84	95.5%	172	91.0%	.193
Divorced/widow	21	7.6%	4	4.5%	17	9.0%	
**Educational level**							
Below secondary	15	5.4%	6	6.8%	9	4.8%	.624
Secondary	41	14.8%	11	12.5%	30	15.9%	
University/above	221	79.8%	71	80.7%	150	79.4%	
**Monthly income**							
<5000 SR	40	14.4%	15	17.0%	25	13.2%	.560
5000–10000 SR	72	26.0%	26	29.5%	46	24.3%	
10000–15000 SR	85	30.7%	27	30.7%	58	30.7%	
15000–20000 SR	50	18.1%	13	14.8%	37	19.6%	
>20000 SR	30	10.8%	7	8.0%	23	12.2%	
**Number of children**							
1 child	37	13.4%	20	22.7%	17	9.0%	.001*
2–3	145	52.3%	52	59.1%	93	49.2%	
4+	95	34.3%	16	18.2%	79	41.8%	

P – Pearson X2 test; * – P<0.05 (significant).

44.4% of children with daily screen time >6 hours were aged 10–12 compared to 29.5% with <5 hours (P=.026, Table 2). Also, 63% of children with daily use >6 hours were males compared to 44.3% of children <5 hours (P=.004). 50% of children with >6 hours of daily screen time had one electronic device compared to 80.6% of those with screen time <5 hours (P=.001). Additionally, 33.8% of children with screen time >6 hours were aged 4–6 years when they received their first device compared to 44.4% of those who used the screen <5 hours daily (P=.007).

**Table 2:** Association between technology use and child's demographic data.

Child data	Total		Screen time				P-value
			≤5 hours		≥6 hours		
	No.	%	No.	%	No.	%	
**Child age**							
6–7	89	32.1%	37	42.0%	52	27.5%	.026*
8–9	78	28.2%	25	28.4%	53	28.0%	
10–12	110	39.7%	26	29.5%	84	44.4%	
**Child gender**							
Male	158	57.0%	39	44.3%	119	63.0%	.004*
Female	119	43.0%	49	55.7%	70	37.0%	
**Does your child have his own electronic device?**							
Yes	232	83.8%	72	81.8%	160	84.7%	.551
No	45	16.2%	16	18.2%	29	15.3%	
**Number of electronic devices**							
One	138	59.5%	58	80.6%	80	50.0%	.001*
Two	76	32.8%	13	18.1%	63	39.4%	
3 or more	18	7.8%	1	1.4%	17	10.6%	
**Child's age when owned first device**							
1–3	44	19.0%	19	26.4%	25	15.6%	.007*$
4–6	86	37.1%	32	44.4%	54	33.8%	
7–10	61	26.3%	16	22.2%	45	28.1%	
>10	41	17.7%	5	6.9%	36	22.5%	

P – Pearson X2 test; $ – Exact probability test; * – P<0.05 (significant).

More than half (56.8%) of the children with <5 hours of screen time daily had a high level of physical activity compared to those who used screens for >6 hours (Table 3). Also, none of the children who used screens for <5 hours had a low level of physical activity *versus* 69.3% of children who used screens for >6 hours (P=.001).

**Table 3:** Relationship between the use of technology and physical activity among typically developing Saudi children aged 6–12 years.

Physical activity	Screen time				P-value
	<5 hours		>6 hours		
	No.	%	No.	%	
**High level**	50	56.8%	0	0.0%	.001*
**Moderate level**	38	43.2%	58	30.7%	
**Low level**	0	0.0%	131	69.3%	

P – Pearson X2 test; $ – Exact probability test; * – P<0.05 (significant).

Among all included predictors, the number of children in the family, having electronic devices, and high amounts of screen time were the most significant predictors of low physical activity (Table 4). Children with more siblings were about five times more likely to have low physical activity. Children with electronic devices were eight times more likely to have low physical activity, and children with >6 hours of daily screen time were four times more likely to have low physical activity.

**Table 4:** Predictors of low-level physical activity level among children of sampled participants.

Predictors	P-value	OR	95% CI	
			Lower	Upper
**Parent age in years**	.480	1.26	0.66	2.40
**Separated/widow parents**	.817	1.36	0.10	18.13
**High education**	.122	3.36	0.72	15.58
**High family income**	.884	1.05	0.53	2.10
**Male child**	.963	1.04	0.17	5.87
**Number of children**	.048*	4.69	1.00	23.72
**Have electronic device**	.046*	8.10	1.10	65.40
**Screen time**	.001*	3.87	2.21	6.78

OR – Odds ratio; CI – Confidence interval; * – P<0.05 (significant).

## Discussion

Our study found that most children spent more than 6 hours on screen, and almost half had a low level of physical activity on the CPAQ. These findings correspond with research in Saudi Arabia [25, 26], Spain and Iran [27, 28], which revealed that the more time a child spends on screen, the less physical activity he or she does. A strong relationship between low levels of physical activity and overweight or obesity was reported in the literature. In line with this, earlier research found a link between growing obesity rates and sedentary behaviours such as playing video games [29]. However, this finding is contrary to previous studies, which have suggested that spending more than 2 hours per day on screens had no association with reduced levels of physical activity [30].

The mean age of parents of low-user children was lower than those of children spending more time on screen, which was inconsistent with a previous study in Spain in which children of younger parents were more likely to use technology [31]. Similarly, prior research found no link between parental age and child technology use [32]. Furthermore, nearly half of the children with more than 6 hours of screen time had 2–3 siblings, compared to the 59.1% of those with less than 5 hours of screen time.

The current study found no significant relationship between parental educational level, marital status, or monthly income and technology use. This finding contradicts a recent study where children from lower-income households used smartphones and tablets more frequently and for longer periods than those from higher-income households [33]. Another significant finding was that most children who remained on their devices for 6 hours or longer were boys. These findings are consistent with Alturki et al. (2020), who discovered that obese boys owned more phones and used mobile screen devices more frequently throughout the week and on weekends [34]. This is also consistent with a previous study reporting boys spent more than five times as much time as girls on the computer and video games [35].

Low physical activity is significantly related to children who use screens for 6 hours or more in the current study. These findings confirm prior research that found significant relationships between higher screen time and lower levels of physical activity [36, 37], indicating the negative influence of sedentary screen time on health among children and adolescents. Also, the analysis revealed that the number of siblings, the ownership of electronic devices, and screen time were the most important predictors of low physical activity. Previous research found that the presence of television in bedroom is linked with more sedentary time and lower levels of physical activity [38, 39]. A possible explanation is that owning a device provides convenient access to games, apps, and applications [40] and competition between the siblings could be a factor contributing to physical inactivity. Low physical activity has been linked to various health problems, including an increased risk of obesity in youngsters. It has been argued that children's screen time is linked to an increased rate of obesity [41–43].

This study contributes to the evidence by identifying an association between various types of technology use and physical activity in typically developing Saudi children aged 6–12 years old. The instrument was a self-reporting questionnaire, and some parts of the questionnaire were very long, which may have influenced the responses of parents and the overall analysis. Furthermore, according to the findings, future studies should use longitudinal and experimental designs, and utilize both subjective and objective measures of screen use.

## Conclusions

The current research was conducted to determine the link between technology use and physical activity in typically developing Saudi children aged 6–12 years. According to our findings, about half of the children had a low level of physical activity. The second key finding was a strong association between the number of children in the family and screen time, with nearly half of children with more than 6 hours of screen time having 2–3 siblings. This study also discovered that youngsters who use screens for 6 hours or more have lower physical activity levels. The study found that the number of siblings, ownership of electronic devices, and screen time were the most important predictors of low physical activity.

Therefore, it is crucial for parents to set limits and boundaries with their children regarding the amount of time they spend each day using technology. At the same time, increasing physical exercise through lifestyle changes involving all the family and scheduling a time each day for outdoor activity, playtime, and minimize screen time is suggested. An implication of this study is that health care providers may regularly screen children and assess them for physical health requirements and provide interdisciplinary care integrated with the community. As a result, health promotion measures aimed at reducing the number of inactive Saudi children should be a top public health goal.

## Data Availability

Data are available upon request.

## Personal thanks

The authors express their thanks to the Deanship of Scientific Research, Deanship of Postgraduate Studies and Scientific Research, King Faisal University for their support and to all the participants for their cooperation.
